# Modeling the Effects of Indoor Passive Smoking at Home, Work, or Other Households on Adult Cardiovascular and Mental Health: The Scottish Health Survey, 2008–2011

**DOI:** 10.3390/ijerph110303096

**Published:** 2014-03-13

**Authors:** Ivy Shiue

**Affiliations:** 1 School of the Built Environment, Heriot-Watt University, Riccarton, EH14 4AS, Edinburgh, Scotland EH14 4AS, UK; E-Mail: I.shiue@hw.ac.uk; Tel.: +44-131-451-4655; Fax: +44-131-451-3161; 2 Owens Institute for Behavioral Research, University of Georgia, 215 S Jackson St. Athens, GA 30602, USA

**Keywords:** housing, public opinion, policy, public health, homeless, social determinants

## Abstract

Passive smoking has contributed increased risks of cardiovascular disease, mental health, and mortality, but the cumulative effects from work or other households were less studied. Therefore, it was aimed to model the effects of indoor passive smoking from own home, work, and other households in a country-wide, population-based setting. Data in the Scottish Health Survey between 2008 and 2011 after the law banning smoking in public places were analyzed. Information including demographics, lifestyle factors, and self-reported cardiovascular disease and mental health was obtained by household interview. Analyses included chi-square test and survey-weighted logistic regression modeling. After full adjustment, it was observed that being exposed to indoor passive smoking, in particular in more than two places of exposure, was significantly associated with risks of stroke, angina, heart attack, abnormal heart rhythms, and GHQ ≥ 12. The significance remained for angina, GHQ ≥ 12 and probably heart attack in never smokers. The cumulative risks also impacted on sleep problems, self-recognition, making decisions, self-confidence, under strain constantly, depressed, happiness and self-worth. The significance remained for sleep problems, self-confidence, under strain constantly, depressed, and happiness in never smokers. Elimination of indoor passive smoking from different sources should still be a focus in future public health programs.

## 1. Introduction

It has been known that passive smoking has contributed increased risks of cardiovascular disease (CVD), mental health, and mortality including in Scotland (from data between 1998 and 2003) [1]. Since the 1980s until recent years [2] there have been several scientific review articles illustrating the worldwide hazard effect of passive smoking on occupants across age groups with various health concerns. The law banning smoking in public places in Scotland has been approved and implemented after scientific evidence was provided [3]. The considerable benefits achieved included air quality improvement, avoidance of adverse health outcomes and broader policy empowerment in Scotland, while the importance of learning from other administrations, and political and professional leadership were also noted until 2008. Further, restriction of passive smoking in households has proved to be protective for children in Scotland [4]. However, the risk effects from different sources other than own home or the cumulative risk effects by counting number of indoor places were less studied. Following the context, therefore, the aim of this work was to model the cumulative risk effects of indoor passive smoking from own home, work, and other households in a country-wide, population-based setting in adults using data from after 2008.

## 2. Experimental Section

### 2.1. Study Sample and Variables

As described elsewhere in detail [5], the Scottish Health Survey [6] has been a country-wide, population-based, multi-year study. It provides a detailed picture of the health of the Scottish population in private households and is designed to make a major contribution to the monitoring of health in Scotland. It is essential for the Scottish Government's forward planning, for identifying gaps in the provision of health services and for identifying which groups are at particular risk of future ill-health. More survey design details, including sample size estimation, can be found online [7]. In the present study, the most recent four years of available data from 2008 to 2011, consisting of four cycles of study cohorts (N = 36,922), after the law banning passive smoking in households were combined for examination. Information including demographics, experiences on being exposed to indoor passive smoking and self-reported health conditions were obtained by household interview. The age range was from 0 to 103, but the adult participants included for the current analysis were those aged between 18 and 103 since information on CV and mental health were obtained in adults only. Study exposures were “whether exposed to passive smoking at home, at work, or at other people’s home (are you regularly exposed to other people's tobacco smoke in any of these places?)” while study outcomes were self-reported CVD, including stroke, angina, heart attack, heart murmur, abnormal heart rhythm, and other heart problems and mental health status by General Health Questionnaire (GHQ). GHQ-12 has been utilized to assess subject’s mental health and psychological distress status [8]. A cut-off of 12 was used to screen people with psychological distress (GHQ score ≥ 12). 

### 2.2. Statistical Analysis

Statistical analyses included chi-square test and logistic regression modeling and models were weighted for survey design. Covariates including age, sex, ethnicity, body mass index (BMI), smoking status, high blood pressure, education level, regular exercise, and alcohol intake were adjusted in the modeling. In the subsequent analysis, never smokers were examined separately. Effects were reported in odds ratios (OR) from general logistic regression models or relative risk ratios (RRR) from multi-level logistic regression models depending on the study outcomes being binary (mainly self-reported CVDs) or ordinal (mainly self-reported mental health status) together with 95% confidence intervals, with *p* < 0.05 considered statistically significant. The statistical software STATA version 13.0 (STATA, College Station, Texas, USA) was used to perform all the analyses. Since this study is only a secondary data analysis based on extracting data from the UK Data Archive website, no further ethics approval is required. The survey was reviewed by an independent Research Ethics Committee and given a favourable opinion by the Cardiff Research Ethics Committee before collecting the primary data [9].

## 3. Results and Discussion

### 3.1. Main Results

Of all included adult participants (N = 27,998), 56.5% were female, 9,087 (32.5%) had education less than high school, while only 6,997 (25.0%) had a normal BMI (18.5–25). Other characteristics were shown in Table 1. In Table 2, the relationships of indoor passive smoking and several self-reported CVDs are shown. After full adjustment (see footnote in the Table 2), it was observed that being exposed to passive smoking in more than two places could largely increase risks of total CVD (OR 1.35, 95%CI 1.11–1.65, *p* = 0.003), stroke (OR 1.88, 95%CI 1.28–2.76, *p* = 0.001), angina (OR 1.50, 95%CI 1.06–2.11, *p* = 0.022), heart attack (OR 1.87, 95%CI 1.29–2.71, *p* = 0.001), abnormal heart rhythms (OR 1.36, 95%CI 1.07–1.74, *p* = 0.013), and GHQ ≥ 12 (OR 1.45, 95%CI 1.26–1.67, *p* < 0.001), but not heart murmur, diabetes, or other heart problems. 

**Table 1:** Characteristics of the whole study cohort and the included adult cohort for analysis.

(N = 36,922)	N (%) or Mean (SD)
Age (range: 0–103)	41.1 (24.3%)
0–29	12,447 (33.7%)
30–59	14,430 (39.1%)
60–79	8,410 (22.8%)
80–99	1,632 (4.4%)
100–103	3 (0.01%)
Sex	
Male	16,693 (45.2%)
Female	20,229 (54.8%)
Adult Cohort (aged 18–103; *n* = 27,998)	
Sex	
Male	12,170 (43.5%)
Female	15,828 (56.5%)
Education	
<high school	9,087 (32.5%)
≥high school	18,848 (67.5%)
Body mass index	
<18.5	283 (1.0%)
18.5–25	6,997 (25.0%)
25–30	8,930 (31.9%)
30–40	6,392 (22.8%)
40 and over	648 (2.3%)
Missing	4,748 (17.0%)
Smoking nowadays	
Current	6,837 (24.2%)
Past	10,252 (36.6%)
Never	10,912 (39.0%)
Secondhand smoking	
Exposed to 0 place	21,268 (76.0%)
Exposed to 1 place	5,388 (19.2%)
Exposed to ≥2 places	1,342 (4.8%)

**Table 2:** Associations of indoor passive smoking and adult CVD.

	Yes *n* (%)	No *n* (%)	Being Exposed in 1 Place			Being exposed in ≥2 Places	
			OR (95%CI)	*p* value		OR (95%CI)	*p* value
Total CVD	10,456 (37.4%)	17,501 (62.6%)	1.25 (1.13–1.37)	<0.001		1.35 (1.11–1.65)	0.003
Stroke	943 (3.4%)	27,041 (96.6%)	1.53 (1.27–1.86)	<0.001		1.88 (1.28–2.76)	0.001
Angina	1,698 (6.1%)	26,272 (93.7%)	1.42 (1.22–1.65)	<0.001		1.50 (1.06–2.11)	0.022
Heart attack	1,177 (4.2%)	26,807 (95.8%)	1.36 (1.14–1.61)	0.001		1.87 (1.29–2.71)	0.001
Heart murmur	1,161 (4.2%)	26,822 (95.8%)	1.19 (1.00–1.42)	0.051		1.17 (0.84–1.64)	0.360
Abnormal heart rhythms	2,140 (7.7%)	25,835 (92.3%)	1.11 (0.97–1.26)	0.130		1.36 (1.07–1.74)	0.013
Other heart problems	833 (3.0%)	27,159 (97.0%)	1.23 (1.00–1.51)	0.051		0.88 (0.55–1.39)	0.581
Diabetes	1,809 (6.5%)	26,181 (93.5%)	1.11 (0.96–1.28)	0.145		1.16 (0.85–1.58)	0.354
GHQ-12 scores ≥ 12	9,009 (35.2%)	16,553 (64.8%)	1.28 (1.18–1.38)	<0.001		1.45 (1.26–1.67)	<0.001

Note: Adjusted for age, sex, ethnicity, body mass index, smoking status, high blood pressure, education level, alcohol intake, and exercise, and survey weighting.

To further explore events within mental health assessment scale (GHQ-12), it was additionally observed that the indoor passive smoking risk effect by accounting the number of exposing places could also impact on sleep problems (more than usual: OR 2.25, 95%CI 1.63–3.11, *p* < 0.001), self- recognition (less than usual: OR 1.83, 95%CI 1.18–2.85, *p* = 0.007), making decisions (less than usual: OR 2.09, 95%CI 1.12–3.90, *p* = 0.020), self-confidence (less than usual OR 2.27, 95%CI 1.64–3.12, *p* < 0.001), under strain constantly (more than usual: OR 1.70, 95%CI 1.22–2.37, *p* = 0.002), depressed (more than usual: OR 2.44, 95%CI 1.76–3.39, *p* < 0.001), happiness (much less than usual: OR, 2.15, 95%CI 1.40–3.30, *p* < 0.001) and self-worth (much less than usual: OR, 1.74, 95%CI 1.18–2.58, *p* = 0.006), in particular when being exposed in more than two places (for details see Table 3). 

**Table 3:** Associations of indoor passive smoking on adult mental health by GHQ-12 items.

GHQ-12 Items	Yes *n* (%)	Being Exposed in 1 Place		Being Exposed ≥2 Places	
		RRR (95%CI)	*p* value	RRR (95%CI)	*p* value
Able to concentrate					
better than usual	779 (2.8%)	reference	n/a	reference	n/a
same as usual	24,033 (86.1%)	1.13 (0.89–1.43)	0.319	0.97 (0.62–1.54)	0.991
less than usual	2,658 (9.5%)	1.43 (1.11–1.85)	0.006	1.59 (0.97–2.61)	0.068
much less than usual	450 (1.6%)	1.94 (1.38–2.72)	<0.001	1.50 (0.81–2.76)	0.197
Lost sleep over worry					
not at all	10,249 (36.7%)	reference	n/a	reference	n/a
no more than usual	13,310 (47.6%)	1.15 (1.06–1.25)	0.001	0.93 (0.80–1.08)	0.341
rather more than usual	3,452 (12.4%)	1.32 (1.17–1.48)	<0.001	1.10 (0.87–1.38)	0.418
much more than usual	927 (3.3%)	1.85 (1.53–2.23)	<0.001	2.25 (1.63–3.11)	<0.001
Felt playing useful part in things					
more than usual	2,098 (7.5%)	reference	n/a	reference	n/a
same as usual	22,872 (82.1%)	1.05 (0.92–1.21)	0.456	1.22 (0.93–1.59)	0.148
less than usual	2,255 (8.1%)	1.40 (1.17–1.67)	<0.001	2.27 (1.63–3.17)	<0.001
much less than usual	645 (2.3%)	1.65 (1.27–2.14)	<0.001	1.83 (1.18–2.85)	0.007
Felt capable of making decisions					
more than usual	1,722 (6.2%)	reference	n/a	reference	n/a
same as usual	24,202 (86.6%)	0.95 (0.81–1.10)	0.471	1.04 (0.79–1.36)	0.806
less than usual	1,684 (6.0%)	1.21 (0.98–1.48)	0.073	1.61 (1.11–2.33)	0.012
much less than usual	328 (1.2%)	1.79 (1.30–2.45)	<0.001	2.09 (1.12–3.90)	0.020
Felt constantly under strain					
not at all	7,544 (27.0%)	reference	n/a	reference	n/a
no more than usual	15,244 (54.6%)	1.05 (0.96–1.15)	0.293	0.93 (0.78–1.12)	0.449
rather more than usual	4,182 (15.0%)	1.21 (1.07–1.36)	0.002	1.07 (0.86–1.35)	0.544
much more than usual	958 (3.4%)	1.50 (1.24–1.82)	<0.001	1.70 (1.22–2.37)	0.002
Felt couldn’t overcome difficulties					
not at all	10,817 (38.7%)	reference	n/a	reference	n/a
no more than usual	14,235 (51.0%)	1.20 (1.11–1.31)	<0.001	1.00 (0.87–1.15)	0.966
rather more than usual	2,257 (8.1%)	1.42 (1.24–1.63)	<0.001	1.48 (1.16–1.90)	0.002
much more than usual	611 (2.2%)	1.98 (1.59–2.49)	<0.001	1.41 (0.96–2.07)	0.080
Able to enjoy day–to–day activities					
more than usual	1,403 (5.0%)	reference	n/a	reference	n/a
same as usual	22,192 (79.4%)	1.03 (0.85–1.24)	0.769	1.15 (0.84–1.58)	0.384
less than usual	3,478 (12.5%)	1.32 (1.07–1.62)	0.010	1.68 (1.216–2.41)	0.005
much less than usual	863 (3.1%)	1.55 (1.19–2.02)	0.001	1.59 (1.00–2.53)	0.051
Been able to face problems					
more than usual	1,212 (4.3%)	reference	n/a	reference	n/a
same as usual	24,314 (87.1%)	0.96 (0.79–1.17)	0.683	0.71 (0.52–0.98)	0.037
less than usual	1,975 (7.1%)	1.36 (1.09–1.70)	0.007	1.17 (0.80–1.72)	0.414
much less than usual	420 (1.5%)	1.44 (1.03–2.01)	0.033	1.05 (0.58–1.88)	0.880
Been feeling unhappy and depressed					
not at all	11,937 (42.7%)	reference	n/a	reference	n/a
no more than usual	11,596 (41.5%)	1.19 (1.10–1.30)	<0.001	1.20 (1.03–1.41)	0.023
rather more than usual	3,492 (12.5%)	1.43 (1.27–1.60)	<0.001	1.80 (1.46–2.22)	<0.001
much more than usual	904 (3.2%)	1.96 (1.62–2.36)	<0.001	2.44 (1.76–3.39)	<0.001
Been losing confidence in self					
not at all	13,269 (47.5%)	reference	n/a	reference	n/a
no more than usual	10,908 (39.1%)	1.12 (1.04–1.22)	0.003	1.09 (0.94–1.26)	0.272
rather more than usual	2,986 (10.7%)	1.39 (1.23–1.56)	<0.001	1.79 (1.45–2.20)	<0.001
much more than usual	765 (2.7%)	1.89 (1.55–2.32)	<0.001	2.27 (1.64–3.12)	<0.001
Been thinking of self as worthless					
not at all	19,055 (68.3%)	reference	n/a	reference	n/a
no more than usual	6,793 (24.3%)	1.17 (1.08–1.28)	<0.001	1.37 (1.17–1.60)	<0.001
rather more than usual	1,494 (5.4%)	1.44 (1.24–1.66)	<0.001	1.94 (1.50–2.52)	<0.001
much more than usual	575 (2.1%)	1.81 (1.42–2.29)	<0.001	1.74 (1.18–2.58)	0.006
Been feeling reasonably happy					
more than usual	2,662 (9.5%)	reference	n/a	reference	n/a
same as usual	22,439 (80.3%)	1.10 (0.97–1.26)	0.141	1.13 (0.89–1.45)	0.311
less than usual	2,283 (8.2%)	1.42 (1.19–1.68)	<0.001	1.70 (1.25–2.29)	0.001
much less than usual	550 (2.0%)	1.83 (1.41–2.37)	<0.001	2.15 (1.40–3.30)	<0.001

Note: Adjusted for age, sex, ethnicity, body mass index, smoking status, high blood pressure, education level, alcohol intake, and exercise, and survey weighting.

In Table 4, by further exploring the variance between urban and rural areas, it was observed that more unexposed people lived in rural areas while more exposed people lived in urban environments. To be specific, the percentage of non-exposure in the city was 72.1% but 83.5% in the remote rural areas. The percentage of being exposed to passive smoking in one place was 22.2% in the city but 13.7% in the remote rural areas while the percentage of being exposed passive smoking in two places was 5.1% in the city but 2.4% in the remote rural areas. Interestingly, the percentage of being exposed to passive smoking in three places did not vary much across urban and rural settings.

**Table 4:** Associations of urbanization levels and indoor passive smoking.

	Not Exposed	Being Exposed in 1 Place	Being Exposed in 2 Places	Being Exposed in 3 Places
City (>125,000 people)	6,594 (72.1%)	2,029 (22.2%)	468 (5.1%)	53 (0.6%)
Urban (>10,000 people)	5,930 (74.3%)	1,643 (20.6%)	356 (4.5%)	51 (0.6%)
Small accessible town	1,861 (76.5%)	438 (18.0%)	120 (4.9%)	14 (0.6%)
Small remote town	1,343 (77.6%)	310 (17.9%)	64 (3.7%)	14 (0.8%)
Rural	2,882 (81.7%)	533 (15.1%)	104 (3.0%)	9 (0.3%)
Remote rural	2,658 (83.5%)	435 (13.7%)	75 (2.4%)	14 (0.4%)

### 3.2. Results on Never Smokers Only

In Table 5, it was shown the relationships of indoor passive smoking and several self-reported CVDs in never smokers only. After full adjustment (see footnote in the table), it was observed that being exposed to passive smoking in more than two places could increase risks of angina (OR 2.43, 95%CI 1.10–5.37, *p* = 0.028), GHQ ≥ 12 (OR 1.63, 95%CI 1.15–2.30, *p* = 0.005) and probably heart attack (OR 2.64, 95%CI 0.99–7.00, *p* = 0.052) which indicated mostly in heart problems.

**Table 5:** Associations of indoor passive smoking and adult CVD in never smokers.

	Yes *n* (%)	No *n* (%)	Being Exposed in 1 Place		Being Exposed in ≥2 Places	
			OR (95%CI)	*p* value	OR (95%CI)	*p* value
Total CVD	1,452 (13.2%)	9,452 (86.8%)	1.24 (1.01–1.52)	0.041	1.75 (0.98–3.14)	0.059
Stroke	258 (2.4%)	10,650 (97.6%)	1.38 (0.89–2.16)	0.151	1.53 (0.41–5.66)	0.523
Angina	442 (4.1%)	10,460 (95.9%)	1.21 (0.86–1.71)	0.270	2.43 (1.10–5.37)	0.028
Heart attack	255 (2.3%)	10,654 (97.7%)	1.37 (0.88–2.14)	0.167	2.64 (0.99–7.00)	0.052
Heart murmur	449 (4.1%)	10,459 (95.9%)	1.24 (0.89–1.71)	0.201	1.29 (0.58–2.87)	0.536
Abnormal heart rhythms	686 (6.3%)	10,220 (93.7%)	1.04 (0.80–1.36)	0.773	2.01 (0.97–4.20)	0.062
Other heart problems	244 (2.2%)	10,665 (97.8%)	1.67 (1.14–2.45)	0.009	0.37 (0.05–2.66)	0.326
Diabetes	579 (5.3%)	10,330 (94.7%)	1.12 (0.85–1.48)	0.422	0.93 (0.45–1.91)	0.838
GHQ-12 scores ≥ 12	3,244 (32.7%)	6,670 (67.3%)	1.12 (0.97–1.29)	0.110	1.63 (1.15–2.30)	0.005

Note: Adjusted for age, sex, ethnicity, body mass index, high blood pressure, education level, alcohol intake, and exercise, and survey weighting.

To further explore events within mental health assessment scale (GHQ-12) in never smokers only, it was additionally observed that the indoor passive smoking risk effect by accounting the number of exposing places could also impact on sleep problems (more than usual: OR 2.53, 95%CI 1.11–5.81, *p* = 0.028), self-confidence (less than usual OR 2.76, 95%CI 1.15–6.64, *p* = 0.023), under strain constantly (more than usual: OR 2.87, 95%CI 1.38–5.95, *p* = 0.005), depressed (more than usual: OR 4.05, 95%CI 1.75–9.37, *p* = 0.001), and happiness (much less than usual: OR, 3.40, 95%CI 1.15–10.08, *p* = 0.027), in particular when being exposed in more than 2 places (for details see Table 6).

**Table 6:** Associations of indoor passive smoking on adult mental health by GHQ-12 items in never smokers.

GHQ-12 Items	Yes *n* (%)	Being Exposed in 1 Place		Being Exposed in ≥2 Places	
		RRR (95%CI)	*p* value	RRR (95%CI)	*p* value
Able to concentrate					
better than usual	779 (2.8%)	reference	n/a	reference	n/a
same as usual	24,033 (86.1%)	1.13 (0.74–1.72)	0.575	1.84 (0.33–10.24)	0.489
less than usual	2,658 (9.5%)	1.21 (0.77–1.89)	0.419	2.72 (0.47–15.63)	0.261
much less than usual	450 (1.6%)	2.39 (1.25–4.57)	0.009	7.05 (1.01–49.04)	0.048
Lost sleep over worry					
not at all	10,249 (36.7%)	reference	n/a	reference	n/a
no more than usual	13,310 (47.6%)	1.09 (0.93–1.27)	0.283	0.96 (0.65–1.41)	0.825
rather more than usual	3,452 (12.4%)	1.37 (1.11–1.70)	0.003	1.28 (0.72–2.26)	0.401
much more than usual	927 (3.3%)	1.51 (1.04–2.19)	0.031	2.53 (1.11–5.81)	0.028
Felt playing useful part in things					
more than usual	2,098 (7.5%)	reference	n/a	reference	n/a
same as usual	22,872 (82.1%)	1.03 (0.80–1.32)	0.826	1.03 (0.53–2.00)	0.932
less than usual	2,255 (8.1%)	1.27 (0.92–1.77)	0.150	1.59 (0.68–3.70)	0.284
much less than usual	645 (2.3%)	1.77 (1.06–2.95)	0.029	2.42 (0.68–8.55)	0.171
Felt capable of making decisions					
more than usual	1,722 (6.2%)	reference	n/a	reference	n/a
same as usual	24,202 (86.6%)	0.95 (0.73–1.23)	0.698	0.95 (0.47–1.89)	0.874
less than usual	1,684 (6.0%)	1.21 (0.84–1.73)	0.301	1.49 (0.61–3.62)	0.379
much less than usual	328 (1.2%)	1.35 (0.68–2.68)	0.395	0.62 (0.08–5.04)	0.655
Felt constantly under strain					
not at all	7,544 (27.0%)	reference	n/a	reference	n/a
no more than usual	15,244 (54.6%)	0.99 (0.84–1.18)	0.943	1.10 (0.67–1.80)	0.705
rather more than usual	4,182 (15.0%)	1.20 (0.96–1.49)	0.102	1.26 (0.68–2.35)	0.458
much more than usual	958 (3.4%)	1.52 (1.05–2.19)	0.026	2.87 (1.38–5.95)	0.005
Felt couldn’t overcome difficulties					
not at all	10,817 (38.7%)	reference	n/a	reference	n/a
no more than usual	14,235 (51.0%)	1.05 (0.91–1.22)	0.505	0.99 (0.67–1.45)	0.944
rather more than usual	2,257 (8.1%)	1.27 (0.98–1.64)	0.071	2.21 (1.22–4.01)	0.009
much more than usual	611 (2.2%)	1.78 (1.14–2.78)	0.011	1.46 (0.50–4.32)	0.484
Able to enjoy day-to-day activities					
more than usual	1,403 (5.0%)	reference	n/a	reference	n/a
same as usual	22,192 (79.4%)	0.96 (0.71–1.30)	0.781	1.74 (0.66–4.56)	0.261
less than usual	3,478 (12.5%)	1.05 (0.74–1.49)	0.801	2.98 (1.07–8.32)	0.037
much less than usual	863 (3.1%)	1.21 (0.74–1.97)	0.441	2.98 (0.77–11.46)	0.112
Been able to face problems					
more than usual	1,212 (4.3%)	reference	n/a	reference	n/a
same as usual	24,314 (87.1%)	1.06 (0.75–1.49)	0.754	1.03 (0.45–2.33)	0.951
less than usual	1,975 (7.1%)	1.30 (0.86–1.99)	0.217	1.86 (0.65–5.31)	0.245
much less than usual	420 (1.5%)	2.06 (1.10–3.86)	0.024	0.69 (0.08–5.99)	0.739
Been feeling unhappy and depressed					
not at all	11,937 (42.7%)	reference	n/a	reference	n/a
no more than usual	11,596 (41.5%)	1.07 (0.92–1.24)	0.383	1.13 (0.76–1.68)	0.553
rather more than usual	3,492 (12.5%)	1.23 (0.99–1.52)	0.059	2.44 (1.53–3.91)	<0.001
much more than usual	904 (3.2%)	1.72 (1.17–2.53)	0.006	4.05 (1.75–9.37)	0.001
Been losing confidence in self					
not at all	13,269 (47.5%)	reference	n/a	reference	n/a
no more than usual	10,908 (39.1%)	0.96 (0.83–1.11)	0.584	1.21 (0.82–1.77)	0.336
rather more than usual	2,986 (10.7%)	1.09 (0.88–1.36)	0.416	2.32 (1.37–3.94)	0.002
much more than usual	765 (2.7%)	1.50 (0.97–2.31)	0.069	2.76 (1.15–6.64)	0.023
Been thinking of self as worthless					
not at all	19,055 (68.3%)	reference	n/a	reference	n/a
no more than usual	6,793 (24.3%)	1.09 (0.93–1.28)	0.275	1.18 (0.77–1.81)	0.450
rather more than usual	1,494 (5.4%)	1.16 (0.87–1.55)	0.326	1.99 (1.01–3.92)	0.047
much more than usual	575 (2.1%)	1.50 (0.91–2.46)	0.113	1.52 (0.55–4.20)	0.421
Been feeling reasonably happy					
more than usual	2,662 (9.5%)	reference	n/a	reference	n/a
same as usual	22,439 (80.3%)	1.03 (0.82–1.30)	0.816	1.16 (0.62–2.17)	0.638
less than usual	2,283 (8.2%)	1.25 (0.91–1.71)	0.170	2.94 (1.41–6.16)	0.004
much less than usual	550 (2.0%)	1.13 (0.65–1.98)	0.657	3.40 (1.15–10.08)	0.027

Note: Adjusted for age, sex, ethnicity, body mass index, high blood pressure, education level, alcohol intake, and exercise, and survey weighting.

### 3.3. Main Findings

In the present study, recent evidence on cumulative effects of indoor passive smoking on CVD and psychological distress from a big Scottish Survey supported by the Scottish Government was provided. The cumulative effects were modeled by counting the number of indoor places. In this context, it was observed that being exposed to indoor passive smoking were significantly associated risks of stroke, angina, heart attack, abnormal heart rhythms, and psychological distress (GHQ ≥ 12), in particular in more than two places of exposure. The significance remained for angina, psychological distress (GHQ ≥ 12) and probably heart attack in never smokers. By exploring mental health specific indicators in detail, it was found that the cumulative risks impacted on sleep problems, self-recognition, making decisions, self-confidence, under strain constantly, depressed, happiness and self-worth. The significance remained for sleep problems, self-confidence, under strain constantly, depressed, and happiness in never smokers. In addition, people living in urban regions tended to be exposed at own home and other households than their counterparts living in the relative rural areas.

### 3.4. Previous Research

Since the 1980s, a few studies have shown the growing concern of passive smoking on Scottish health [10] including cardiorespiratory health [11,12], middle ear under pressure and effusion in children [13], coronary heart disease markers [14,15], lung function [16], and intermittent claudication in the middle-aged [17]. In addition to CVD such as stroke or coronary heart disease, less attention was paid to other forms of heart problems such as angina, heart attack, and abnormal heart rhythms in Scotland. However, previous research in the USA [18], Canada [19], and Greece [20] have indicated that clean indoor air environment could help reduce burden of angina and indoor passive smoking could impair symptomatic improvement in patients with chronic angina undergoing enhanced external counterpulsation [21]. Mostly passive smoking comes from sidestream smoke emitted from the burning tip of the cigarette and sidestream smoke is hazardous because it contains high concentrations of ammonia, benzene, nicotine, carbon monoxide, and many carcinogens [22]. Smoking could elicit both acute and chronic cardiac and vascular events due to the multiplicity of mechanisms involved hematological, neurohormonal, metabolic, hemodynamic, molecular genetic and biochemical pathways [23]. On the other hand, a few review articles have been supporting the view that smoking could act negatively on the heart causing atherosclerotic coronary alterations, focal myocardial lesions and arrhythmias [24,25]. Of note, these previous studies were mostly with rather small study sample.

### 3.5. Strengths and Limitations

The strength of this study lies in the very large study sample from across Scotland and could provide statistical power in modeling risk effects of indoor passive smoking. This is for the first time to examine the cumulative risk effect by counting the number of indoor places of exposure including own home, at work, and in other households. However, one limitation is that it was unable to assess the duration and the amount of being exposed to passive smoking from different indoor places including the historical records in the past during the childhood, adolescence, and/or early adulthood. Therefore, future research keeping the strengths and overcoming the limitations is recommended. 

## 4. Conclusions

In summary, recent evidence has shown the cumulative risk effects of indoor passive smoking on CV and mental health in big Scottish population cohorts after the law banning smoking in public places since 2008. It has been known notoriously that being exposed to passive smoking is harmful for human health and a serious public health concern across the globe. Although the chance of exposure at work has been lessened due to the introduction of policy regulation, the risk effects from own home and other people’s households seem to have persisted and affected human health including CVD (mostly heart problems) and mental health. Therefore, elimination of passive smoking from different indoor places should still be a top priority in future public health and perhaps housing policy. In particular, restriction of passive smoking in the households has proved to be protective for children in Scotland [3]. Wider implementation on restriction of indoor passive smoking to adults should therefore be further considered.
